# Causal relationship between PCSK9 inhibitor and autoimmune diseases: a drug target Mendelian randomization study

**DOI:** 10.1186/s13075-023-03122-7

**Published:** 2023-08-14

**Authors:** Weijia Xie, Jiaxin Li, Hao Du, Jian Xia

**Affiliations:** 1 Department of Neurology, Xiangya Hospital, Central South University, 87# Xiangya Road, Changsha, Hunan China; 2 Clinical Research Center for Cerebrovascular Disease of Hunan Province, Central South University, Changsha, Hunan China; 3 National Clinical Research Center for Geriatric Disorders, Xiangya Hospital, Central South University, Changsha, Hunan China

**Keywords:** Drug-target Mendelian randomization, PCSK9, HMGCR, Autoimmune disease, Immune regulation

## Abstract

**Background:**

In addition to decreasing the level of cholesterol, proprotein convertase subtilis kexin 9 (PCSK9) inhibitor has pleiotropic effects, including immune regulation. However, the impact of PCSK9 on autoimmune diseases is controversial. Therefore, we used drug target Mendelian randomization (MR) analysis to investigate the effect of PCSK9 inhibitor on different autoimmune diseases.

**Methods:**

We collected single nucleotide polymorphisms (SNPs) of PCSK9 from published genome-wide association studies statistics and conducted drug target MR analysis to detect the causal relationship between PCSK9 inhibitor and the risk of autoimmune diseases. 3-Hydroxy-3-methylglutaryl-assisted enzyme A reductase (HMGCR) inhibitor, the drug target of statin, was used to compare the effect with that of PCSK9 inhibitor. With the risk of coronary heart disease as a positive control, primary outcomes included the risk of systemic lupus erythematosus (SLE), rheumatoid arthritis (RA), myasthenia gravis (MG), multiple sclerosis (MS), asthma, Crohn’s disease (CD), ulcerative colitis (UC), and type 1 diabetes (T1D).

**Results:**

PCSK9 inhibitor significantly reduced the risk of SLE (OR [95%CI] = 0.47 [0.30 to 0.76], *p* = 1.74 × 10^−3^) but increased the risk of asthma (OR [95%CI] = 1.15 [1.03 to 1.29], *p* = 1.68 × 10^−2^) and CD (OR [95%CI] = 1.38 [1.05 to 1.83], *p* = 2.28 × 10^−2^). In contrast, HMGCR inhibitor increased the risk of RA (OR [95%CI] = 1.58 [1.19 to 2.11], *p* = 1.67 × 10^−3^), asthma (OR [95%CI] = 1.21 [1.04 to 1.40], *p* = 1.17 × 10^−2^), and CD (OR [95%CI] = 1.60 [1.08 to 2.39], *p* = 2.04 × 10^−2^).

**Conclusions:**

PCSK9 inhibitor significantly reduced the risk of SLE but increased the risk of asthma and CD. In contrast, HMGCR inhibitor may be a risk factor for RA, asthma, and CD.

**Supplementary Information:**

The online version contains supplementary material available at 10.1186/s13075-023-03122-7.

## Background

The incidence of autoimmune diseases accounts for about 5–9% of the global population, and their treatment is still a thorny clinical problem that imposes a great burden on society and families [1]. The main therapeutic direction of autoimmune diseases is non-disease-specific immune regulation and immunosuppression, which typically necessitates long-term steroid therapy and causes severe side effects with little improvement in symptoms [2, 3]. Therefore, it is imperative to explore the underlying pathogenesis and identify novel therapeutic targets to more effectively prevent and treat autoimmune diseases [3]. Studies have shown that patients with autoimmune diseases, such as systemic lupus erythematosus (SLE) and rheumatoid arthritis (RA), are more susceptible to dyslipidemia [4–6]. Additionally, lipid-lowering therapy has been found to improve the prognosis of SLE [7, 8]. Consequently, dyslipidemia may be implicated in the pathogenesis and progression of autoimmune diseases, but the effects of various lipid-lowering drugs on autoimmune diseases warrant further investigation.

Proprotein convertase subtilis kexin 9 (PCSK9) is a serine protease that plays a significant role in the regulation of low-density lipoprotein cholesterol (LDL-C) metabolism and has emerged as a critical target for cholesterol-lowering therapy [9]. Although the protective effects of PCSK9 inhibitors (PCSK9i) in cardiovascular diseases (CVD) have been widely established, the impact of PCSK9 on autoimmune diseases remains uncertain. Clinical evidence suggests that the serum PCSK9 level is positively correlated with disease activity and damage in patients with SLE [10, 11] but is decreased in those with RA [12]. Currently, there is no direct evidence to support the role PCSK9 plays in autoimmune diseases. However, some researches have suggested a possible link between PCSK9 and the immune system. It is important to note that PCSK9i have potential pleiotropic effects beyond lowering lipids. Compared to traditional lipid-lowering drugs such as statins (3-hydroxy-3-methylglutaryl coenzyme A reductase inhibitor, HMGCRi), PCSK9i can regulate the immune function by suppressing dendritic cell (DC)-mediated T cell activation [13]. Moreover, PCSK9 promotes the secretion of inflammatory cytokines by monocyte-macrophages via TLR4/NF-κB and other inflammatory pathways, which can be inhibited by PCSK9i [14, 15]. These findings suggest that PCSK9i may be involved in the pathogenesis of autoimmune diseases through pathways other than lipid-lowering. However, the effects of PCSK9i on various autoimmune diseases may be inconsistent, necessitating further exploration.

Drug target Mendelian randomization (MR) analysis takes the genetic variation that simulates the pharmacological inhibition of pharmacogenetic targets as the instrumental variables. By conducting regression analysis, it can elucidate the effects of long-term drug use and strengthen the causal inference of the potential impact of these drug gene targets on autoimmune diseases [9, 16]. In this study, we collected the recently published genome-wide association study (GWAS) summary-level statistics to investigate the causal relationship between the genetically predicted inhibition of PCSK9 and HMGCR and autoimmune diseases, including SLE, RA, myasthenia gravis (MG), multiple sclerosis (MS), asthma, Crohn’s disease (CD), ulcerative colitis (UC), and type 1 diabetes (T1D), by conducting a drug-targeted MR analysis.

## Methods

### Selection of PCSK9 and HMGCR instrumental variables

**Fig. 1:**
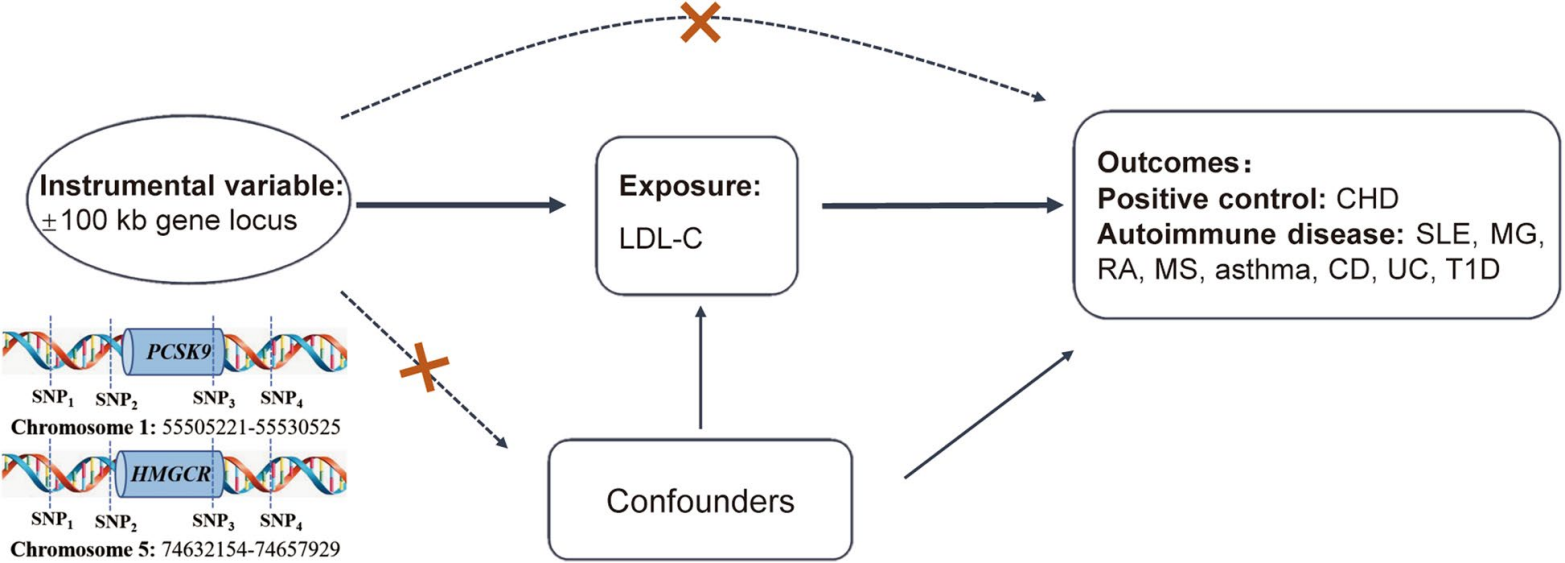
Research overview and design of drug target Mendelian randomization analysis. PCSK9 and HMGCR inhibitors have been widely used to reduce the risk of coronary heart disease (CHD). So, we selected CHD as a positive control. In order to verify the existence of causal correlation, it is necessary to meet the conditions as follows: (1) the instrumental variables are not related to the confounders (dashed line), (2) the instrumental variables are related to the exposure factor (solid line), and (3) the instrumental variables are not directly related to the outcome (dashed line). LDL-C, low-density lipoprotein cholesterol; PCSK9, proprotein convertase subtilisin/kexin type 9; HMGCR, 3-hydroxy-3-methylglutaryl-coenzyme A reductase; CHD, coronary heart disease; SLE, systemic lupus erythematosus; RA, rheumatoid arthritis; MG, myasthenia gravis; MS, multiple sclerosis; CD, Crohn’s disease; UC, ulcerative colitis; T1D, type 1 diabetes

The summary data of LDL-C is from a GWAS summary statistics containing 440,546 European individuals [17]. By obtaining instrumental variables that can target PCSK9 and HMGCR to reduce LDL-C, it can be used to simulate the effects of PCSK9 inhibitor and HMGCR inhibitor (statins) [17]. The instrumental variables select single nucleotide polymorphism (SNPs) that are located within ± 100kb of PCSK or HMGCR loci and related to LDL-C level (Fig. 1). In order to avoid the effect of strong linkage disequilibrium (LD) on the results, the threshold of LD was set (*r*^2^ < 0.3). Finally, 32 significant SNPs of PCSK9 and 12 significant SNPs of HMGCR were retained (Additional file 1: Table S1). Using the summary data of another GWAS study involving 94,595 people of European descent, the instrumental variables of PCSK9 and HMGCR (Additional file 1: Table S2) were obtained again using the above method for repeated analysis to ensure the stability of the results [18].

### Source of outcomes

We used nine diseases as the results of the drug target MR analysis, of which coronary heart disease (CHD) was a positive control dataset. The datasets were all from the European population. The CHD dataset was obtained from a GWAS summary statistics containing 42,096 cases and 99,121 controls [19]. In addition, we also collected the summary dataset of the GWAS of SLE [20], MG [21], RA [22], MS [23], asthma [24], CD [25], UC [26], and TID [27] as the primary outcomes.

### Data analysis

The inhibitors of PCSK9 and HMGCR have been widely used in the treatment of CHD. Therefore, we use the summary data of the GWAS of CHD as the positive control of the results to verify the effectiveness of the instrumental variables. First, we harmonized the exposure-related drug targeting instrumental variables with the outcome datasets, and then use MR Egger, weighted median, inverse variance weighted (IVW), simple mode, weighted mode, and MR-PRESSO for analysis, of which the IVW method is the most commonly used method [28]. The heterogeneity test was carried out by MR Egger and IVW methods. Cochrane’s *Q* value was used to evaluate the heterogeneity of genetic tools and *p* > 0.05 showed that there was no significant heterogeneity. The MR Egger regression equation was used to evaluate the horizontal pleiotropy of the genetic tool, and *p* > 0.05 showed that there was no horizontal pleiotropy [29].

The hypothesis of MR requires that SNP is not directly related to the outcome (Fig. 1). Therefore, the online website PhenoScanner (http://www.phenoscanner.medschl.cam.ac.uk/) was used to find the traits directly related to the tool variable SNP, excluding SNP related to CHD, SLE, MG, RA, MS, asthma, CD, UC, and T1D. Sensitivity analysis was performed again after removing the outlier through the MR-PRESSO test. In order to ensure that our results will not be significantly affected by a certain SNP, we used the leave-one-out method to remove each SNP in turn and compared the results of the IVW method with all variants. The data analysis was performed on R version 4.0.2 using MRPRESSO and TwoSampleMR packages [29, 30].

## Results

### Positive control analysis

**Fig. 2:**
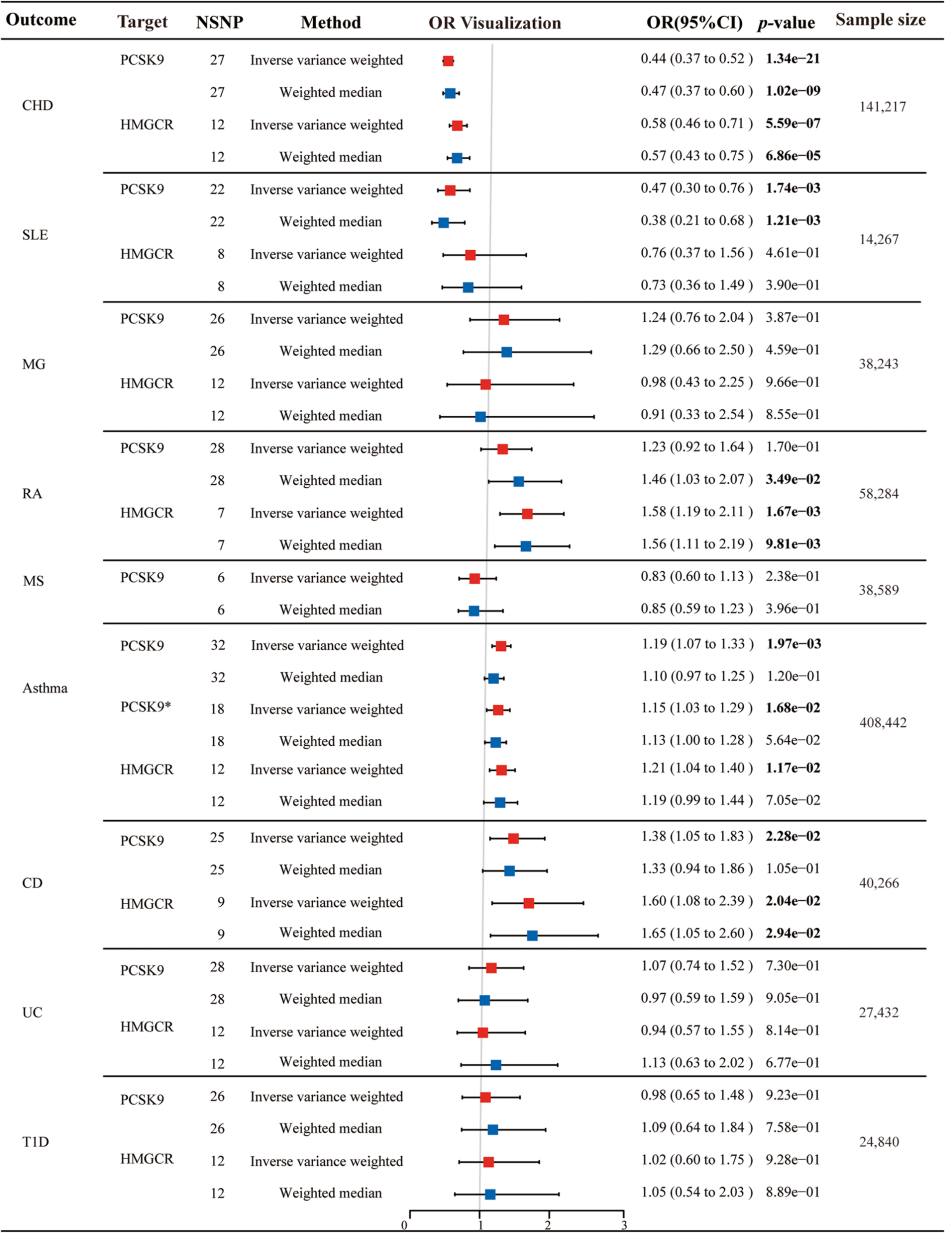
The effect of PCSK9 and HMGCR inhibitor on coronary heart disease and autoimmune diseases. Asterisk (*) represents the linkage disequilibrium (LD) parameter in the selection of instrumental variables changes from *r*^2^ < 0.3 to *r*^2^ < 0.1. NSNP, number of single nucleotide polymorphisms; OR, odds ratio; CI, confidence interval; PCSK9, proprotein convertase subtilisin/kexin 9; HMGCR, 3-hydroxy-3-methylglutaryl coenzyme A reductase; CHD, coronary heart disease; SLE, systemic lupus erythematosus; RA, rheumatoid arthritis; MG, myasthenia gravis; MS, multiple sclerosis; CD, Crohn’s disease; UC, ulcerative colitis; T1D, type 1 diabetes

As expected, the results of the IVW method demonstrated that PCSK9i significantly reduced the risk of CHD (OR [95%] = 0.44 [0.37 to 0.52], *p* = 1.34 × 10^−21^), which was similar to the effect of HMGCRi (OR [95%] = 0.58 [0.46 to 0.71], *p* = 5.59 × 10^−7^) (Fig. 2). The results of MR Egger, simple mode, weighted mode, and MR-PRESSO are shown in Additional file 1: Table S3. Similar results were obtained by repeating the analysis with another GWAS dataset (Additional file 1: Table S4).

### The causal relationship between gene-simulated inhibition of PCSK9 and HMGCR and autoimmune diseases

Genetically predicted inhibition of PCSK9 had an obvious protective effect on SLE in both the IVW method (OR [95%] = 0.47 [0.30 to 0.76], *p* = 1.74 × 10^−3^) and weighted median method (OR [95%] = 0.38 [0.21 to 0.68], *p* = 1.21 × 10^−3^), while inhibition of HMGCR did not reach statistical significance (IVW: *p* = 0.46; weighted median: *p* = 0.39) (Fig. 2). The results of other MR analysis methods were shown in Additional file 1: Table S3.

Additionally, genetically predicted inhibition of HMGCR had a positive correlation effect on the risk of RA (IVW: OR [95%] = 1.58 [1.19 to 2.11], *p* = 1.67 × 10^−3^), while inhibition of PCSK9 had no effect on the risk of RA (IVW: *p* = 0.17). Besides, both inhibition of PCSK9 and HMGCR could significantly increase the risk of asthma (PCSK9: IVW: OR [95%] = 1.19 [1.07 to 1.33], *p* = 1.97 × 10^−3^; HMGCR: IVW: OR [95%] = 1.21 [1.04 to 1.40], *p* = 1.17 × 10^−2^) and CD (PCSK9: IVW: OR [95%] = 1.38 [1.05 to 1.83], *p* = 2.28 × 10^−2^; HMGCR: IVW: OR [95%] = 1.60 [1.08 to 2.39], *p* = 2.04 × 10^−2^)(Fig. 2). However, neither PCSK9i nor HMGCRi was significantly associated with the risk of MG, MS, UC, and T1D (Additional file 1: Table S3 and Fig. 2).

Furthermore, we used another GWAS dataset to conduct the repeated analysis and reached similar conclusions (Additional file 1: Table S4).

### Sensitivity analysis

**Fig. 3:**
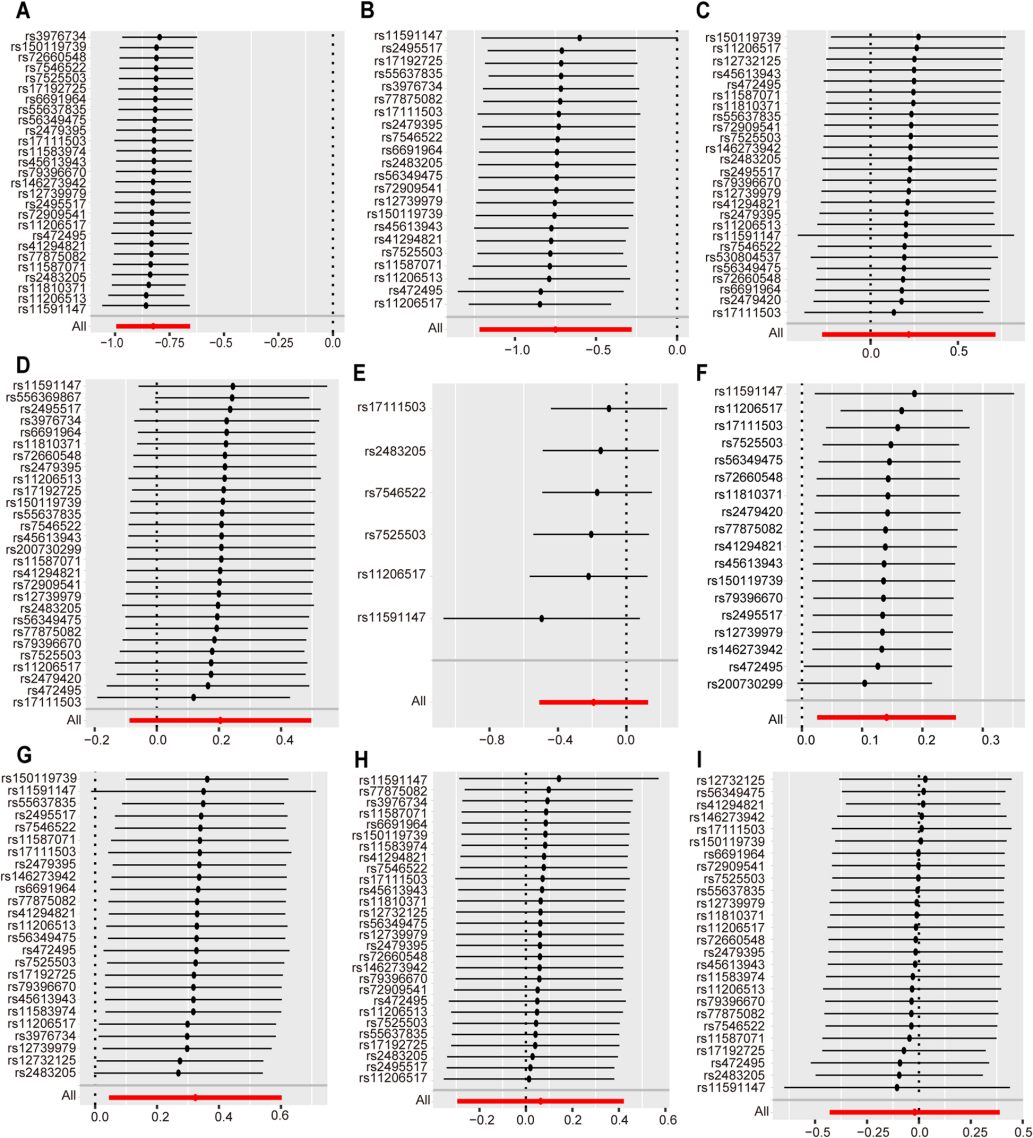
Sensitivity analysis of PCSK9 on coronary heart disease and autoimmune diseases. Leave-one-out analysis of PCSK9 on **A** CHD, **B** SLE, **C** MG, **D** RA, **E** MS, **F** asthma, **G** CD, **H** UC, and **I** T1D. The leave-one-out method is used to evaluate the excessive impact of a single SNP on MR analysis if the comprehensive effect of the remaining SNPs is consistent with the main effect after removing one SNP. SNP, single nucleotide polymorphisms; PCSK9, proprotein convertase subtilisin/kexin type 9; CHD, coronary heart disease; SLE, systemic lupus erythematosus; RA, rheumatoid arthritis; MG, myasthenia gravis; MS, multiple sclerosis; CD, Crohn’s disease; UC, ulcerative colitis; T1D, type 1 diabetes

**Fig. 4:**
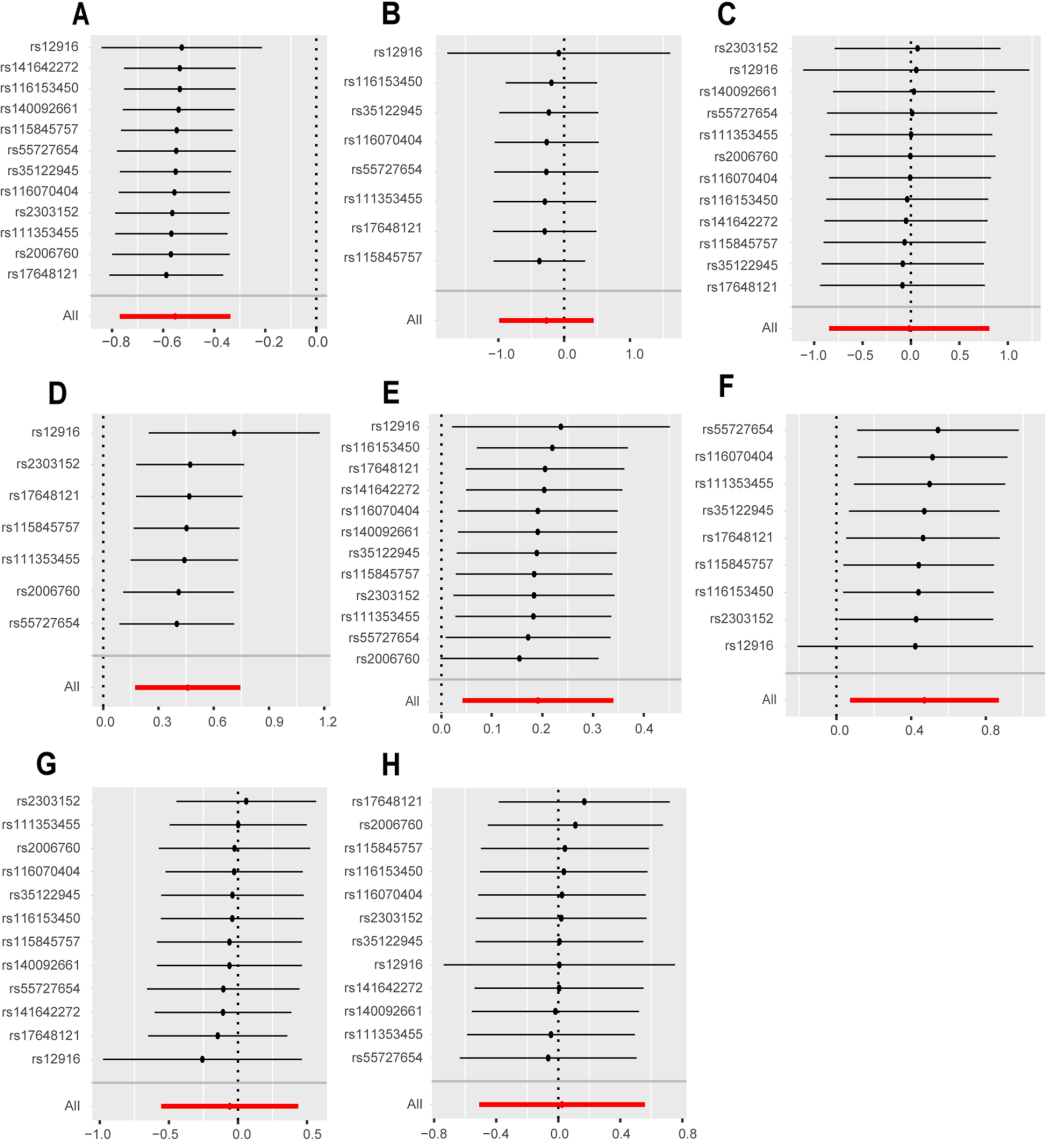
Sensitivity analysis of HMGCR on coronary heart disease and autoimmune diseases. Leave-one-out analysis of HMGCR on **A** CHD, **B** SLE, **C** MG, **D** RA, **E** asthma, **F** CD, **G** UC, and **H** T1D. The leave-one-out method is used to evaluate the excessive impact of a single SNP on MR analysis if the comprehensive effect of the remaining SNPs is consistent with the main effect after removing one SNP. SNP, single nucleotide polymorphisms; HMGCR, 3-hydroxy-3-methylglutaryl-coenzyme A reductase; CHD, coronary heart disease; SLE, systemic lupus erythematosus; RA, rheumatoid arthritis; MG, myasthenia gravis; CD, Crohn’s disease; UC, ulcerative colitis; T1D, type 1 diabetes

Cochrane’s *Q* and MR Egger regression equation were used to evaluate the level of heterogeneity and horizontal pleiotropy (Additional file 1: Tables S5-S6). We revealed significant heterogeneity (*p* = 4.53x10^-3^) and horizontal pleiotropy (*p* = 1.49x10^-2^) when we investigated the causality between the inhibition of PCSK9 and asthma (Additional file 1: Table S5). Notably, the random-effect IVW method we adopted can eliminate the bias caused by heterogeneity in the results [31]. Furthermore, employing more stringent criteria for SNP selection effectively reduced both heterogeneity and horizontal pleiotropy in instrumental variables. Therefore, to obtain more reliable results, we performed the MR analysis again using the instrumental variables selected under stricter criteria (changing the LD parameter from *r*^2^ < 0.3 to *r*^2^ < 0.1) (Additional file 1: Table S1). Encouragingly, our updated analysis still demonstrated that the inhibition of PCSK9 was associated with a significantly increased risk of asthma (IVW: OR [95%] = 1.15 [1.03 to 1.29], *p* = 1.68 × 10^−2^) (Additional file 1: Table S3 and Fig. 2), and this result exhibited no significant heterogeneity or horizontal pleiotropy (Additional file 1: Table S5). Besides, the results of sensitivity analysis showed that there were no heterogeneity and horizontal pleiotropy in all other outcomes (*p* > 0.05) (Additional file 1: Table S5). The leave-one-out method showed that there would be no significant difference in the results after removing any SNP for CHD and autoimmune diseases (Figs. 3 and 4). In addition, another LDL-C-related GWAS was used to re-select PCSK9 and HMGCR instrumental variables, and repeating the above solutions showed that a stable outcome could be obtained (Additional file 1: Table S6).

## Discussion

The inhibition of PCSK9 by antibodies represents a novel principle of lowing the level of LDL-C, which has been widely proved to be beneficial for the prevention and treatment of CVD in clinical practice [32, 33]. In addition to its effects on LDL-C levels, PCSK9i has potential pleiotropic effects, including enhancing the response of tumors to immune checkpoint therapy, suppressing platelet activation and thrombosis, and reducing cell apoptosis [34–36]. Recently, the role of PCSK9 in inflammation and immunity, especially in the pathogenesis of autoimmune diseases, has received increasing attention [13, 37]. Nevertheless, no study has comprehensively investigated the causal relationship between PCSK9i and the risk of autoimmune diseases. After performing a drug target MR analysis, we found that PCSK9i could significantly reduce the risk of SLE, but may be a risk factor of asthma and CD. Our finding may help us to have a deeper understanding of the inflammatory effect of PCSK9i, provide clues for the possible side effects of PCSK9i, and offer theoretical guidance for the selection of lipid-lowering strategies.

Excitingly, our results showed that PCSK9i had a clear protective effect on SLE. SLE is a disease with a relapsing–remitting autoimmune course, affecting almost every organ in the human body [38]. Compared with healthy controls, patients with SLE have higher levels of serum PCSK9, particularly those with thickening of carotid intima-media thickness (cIMT) [39]. Elevated levels of PCSK9 are also detected in the serum of SLE patients with higher disease activity and severer damage, suggesting a close association between PCSK9 and the status of SLE [10, 11]. However, the mechanism by which PCSK9 participates in the occurrence and progression of SLE remains unclear. In our study, PCSK9i had a protective effect on the risk of SLE, while HMGCRi did not achieve a similar effect, suggesting that PCSK9i may reduce the risk of SLE through pathways other than lipid-lowering. This also implied that changes in blood lipids were not associated with the risk of SLE, which was consistent with the conclusions drawn from recent MR studies [40, 41]. A case–control study shows oxidized LDL (oxLDL)-induced activation and maturation of DC are stronger in patients with SLE than in healthy controls, and this effect can be suppressed by the inhibition of PCSK9, indicating that PCSK9 may be involved in the progression of SLE by affecting inflammatory and immune-related pathways [10]. Sufficient evidence manifests that patients with SLE have a significantly elevated risk of CVD and a tendency for atherosclerosis (AS) progression, which can only partially be explained by traditional CVD risk factors, such as age and blood lipids parameters, including triglyceride (TG), total cholesterol (TC), and LDL-C [42–44]. Widespread immune dysfunction, vascular endothelial injury, and systemic inflammatory responses may participate in the process of SLE-related CVD [44, 45]. The level of serum PCSK9 in SLE patients with AS is higher and proportional to C-reactive protein (CRP) compared to AS-free SLE patients, which cannot be fully explained by traditional CVD risk factors, suggesting that inflammatory imbalances caused by PCSK9 play a critical role in SLE-related AS [39]. Additionally, thrombotic events occur more frequently in SLE patients than in the general population, which is related to hypertension, neutrophil extracellular traps, and antiphospholipid antibodies(aPL) [46–48]. Antibodies to CD36, a platelet glycoprotein, are associated with thrombosis, and anti-CD36 is highly prevalent in patients with aPL and with a trend to being more common in patients with recurrent thrombosis [49–51]. PCSK9 enhances platelet activation and thrombosis by binding to platelet CD36 and activating the p38MAPK signaling pathway [35]. PCSK9i may also affect the occurrence and progression of SLE by inhibiting platelet activation and thrombosis in addition to lipid-lowering and immune and inflammatory regulation.

In contrast, we found HMGCRi significantly increased the risk of RA, but PCSK9i did not affect the incidence of RA, indicating the side effects of PCSK9i may be less than that of HMGCRi. The lipid profile of RA patients shows characteristic changes in different disease stages. Compared with the non-RA cohort, TC and LDL-C levels in RA patients decrease significantly in the 5 years prior to onset and are not attributable solely to lipid-lowering therapy [52]. TC and LDL-C levels are elevated in pre-stage and early-stage RA patients but decrease in highly active, therapy-resistant RA patients, which may be related to the lipid-lowering effects of RA-related systemic inflammation [53, 54]. The systemic inflammation caused by RA would further aggravate the dyslipidemia in RA patients and affect the prognosis of the disease [54]. Therefore, inflammatory response is a key factor affecting the lipid profile of RA patients. Our study found that PCSK9i, which has a role in regulating inflammation, was not associated with an increased risk of RA. A previous case–control study reported that PCSK9 serum concentration was downregulated in patients with RA [12]. However, a recent study shows that the level of PCSK9 is elevated and positively correlated with the level of CRP and disease activity in RA patients [55]. Furthermore, a reduced level of PCSK9 is associated with increased response to treatment and remission in RA patients treated with conventional synthetic disease-modifying anti-rheumatic drugs [55]. Patients with lower PCSK9 levels at baseline also have a better response to anti-tumor necrosis factor-α (anti-TNF-α) therapy, possibly due to the pro-inflammatory effect of PCSK9 in stimulating macrophages and synoviocytes to produce pro-inflammatory cytokines, including TNF-α, interleukin-1β (IL-1β), and monocyte chemoattractant protein-1(MCP-1) [56]. This pro-inflammatory effect of PCSK9 can be suppressed by the inhibition of PCSK9, confirming the pro-inflammatory role of PCSK9 in RA [56]. Therefore, the anti-inflammatory effect of PCSK9i may reduce the risk of RA caused by lipid-lowering and help relieve dyslipidemia in RA.

However, we found PCSK9i could significantly increase the risk of CD and asthma, indicating potential long-term side effects of PCSK9i. As for inflammatory bowel disease (IBD), we found both HMGCRi and PCSK9i increased the risk of CD but had no association with the risk of UC. The lipid profile of patients with IBD indicates lower serum TC and LDL-C levels are associated with a higher incidence of CD but not UC [57]. Serum TC and LDL-C levels in patients with active CD are significantly lower than those in healthy controls [58]. Therefore, the lipid-lowering effect of HMGCRi and PCSK9i may increase the risk of the development of CD. Besides, our study also detected that PCSK9i could increase the risk of asthma, which was consistent with the previous MR studies [59–61]. Interestingly, we found that HMGCRi had a similar effect, suggesting PCSK9i and HMGCRi may increase the risk of asthma through a lipid-lowering effect. However, previous meta-analysis studies have reported that serum LDL-C levels in patients with asthma are higher than in healthy controls [62, 63]. This discrepancy may be due to the limitations of MR, which can only reflect the effects of lifetime exposure, and not explore possible changes across ages without continuous assessment. Therefore, while drug target MR provides an indication of the effect direction, it may not directly predict the magnitude of the pharmacological effect of a drug on its target.

Moreover, our study did not observe a causal relationship between the inhibition of PCSK9 and the risk of T1D, MG, and MS. A precious study suggests the T allele of *PCSK9* rs1159147, which is associated with lower LDL levels, is significantly related to an increased risk of T1D [59]. Our study included more SNP of *PCSK9* related to the level of LDL and reached a more comprehensive conclusion that PCSK9i-mediated LDL level reduction was not associated with the risk of T1D. Both MG and MS are autoimmune diseases of the nervous system. Clinical evidence suggests that statin can induce and aggravate MG [64, 65], while studies on PCSK9 and MG are limited. Therefore, the formulation of a lipid-lowering strategy should be considered comprehensively to reduce drug-induced MG. MS is a chronic inflammatory and autoimmune demyelinating disease of the central nervous system (CNS) that can cause neuronal damage and disabling neurological deficits [66]. The influence of cholesterol and its metabolites on the pathophysiology of MS has been a topic of interest. Patients with MS have higher levels of serum TC than healthy controls [67]. GWAS also shown genetic overlap between MS and CVD risk factors, including LDL [68]. Nevertheless, cholesterol is essential in the CNS because it is a component of cell membranes and myelin and is required for synapse and dendrite formation and axonal guidance [69]. In animal models, statins alleviate the severity of MS [70, 71]. Inhibition of PCSK9 does not improve MS symptoms, although it reduces the level of circulating LDL [72]. Thus, the impact of circulating blood cholesterol levels in the development and progression of MS remains controversial. Although we did not find a significant association between inhibition of PCSK and the risk MS, previous drug target MR analysis suggests that inhibition of PCSK9 is a protective factor for MS [60, 61]. Further clinical trials and mechanism studies are needed to better understand the role of PCSK9 in the pathophysiology of MS.

It must be admitted that our study has several inescapable limitations. Firstly, MR analysis cannot substitute for clinical trials in the objective world since it is only a method used to analyze the causal relationship between exposure and outcome. Further studies are needed to confirm the association between the inhibition of PCSK9 and the risk of autoimmune diseases. Besides, we only conducted MR analysis in the European population due to the insufficient GWAS data resources. The efficacy and side effects of PCSK9i may differ among different populations due to the genetic heterogeneity among various ethnic groups. Therefore, future studies should conduct subgroup analyses in diverse populations to obtain a more comprehensive conclusion.

## Conclusions

After performing a drug target MR analysis, we found genetically predicted inhibition of PCSK9 significantly reduced the risk of SLE but increased the risk of asthma and CD. In contrast, genetically predicted inhibition of HMGCR may be a risk factor of RA, asthma, and CD.

### Supplementary Information

**Additional file 1.** Supplementary tables.

## Data Availability

The authors confirm that the data supporting the findings of this study are available within the article and its supplementary materials.

## Abbreviations

PCSK9: Proprotein convertase subtilis kexin 9
MR: Mendelian randomization
SNP: Single nucleotide polymorphism
HMGCR: 3-Hydroxy-3-methylglutaryl-assisted enzyme A reductase
SLE: Systemic lupus erythematosus
RA: Rheumatoid arthritis
MG: Myasthenia gravis
MS: Multiple sclerosis
CD: Crohn’s disease
UC: Ulcerative colitis
T1D: Type 1 diabetes
LDL-C: Low-density lipoprotein cholesterol
CVD: Cardiovascular diseases
DC: Dendritic cell
GWAS: Genome-wide association study
CHD: Coronary heart disease
IVW: Inverse variance weighted
cIMT: Carotid intima-media thickness
AS: Atherosclerosis
TG: Triglyceride
TC: Total cholesterol
CRP: C-reactive protein
aPL: Antiphospholipid antibodies
IL: Interleukin
MCP-1: Monocyte chemoattractant protein-1
IBD: Inflammatory bowel disease
CNS: Central nervous system
PCSK9i: PCSK9 inhibitor
HMGCRi: 3-hydroxy-3-methylglutaryl coenzyme A reductase inhibitor
LD: Linkage disequilibrium
TNF-α: Tumor necrosis factor-α
