# Supplementary material for: Causal relationship between PCSK9 inhibitor and autoimmune diseases: a drug target Mendelian randomization study
Source: Arthritis Res Ther. 2023 Aug 14;25:148. doi: 10.1186/s13075-023-03122-7 (PMC10424393; doi:10.1186/s13075-023-03122-7)
Supplement: Supplementary file 1 — Additional file 1. Supplementary tables. [file 13075_2023_3122_MOESM1_ESM.docx]

**Supplementary Table1** The detail of instrumental variable corresponding to PCSK9 and HMGCR.

|  | SNP | chr | pos | Beta | SE | *p*-value | Effect alleles | Other alleles |
| --- | --- | --- | --- | --- | --- | --- | --- | --- |
| PCSK9 | rs6691964 | 1 | 55433978 | -0.02347 | 0.003584 | 5.80E-11 | A | G |
| PCSK9 | rs2495517* | 1 | 55448842 | 0.017755 | 0.002579 | 5.80E-12 | G | A |
| PCSK9 | rs146273942* | 1 | 55453841 | -0.05389 | 0.007224 | 8.70E-14 | A | G |
| PCSK9 | rs55637835 | 1 | 55466303 | -0.01871 | 0.003248 | 8.40E-09 | T | C |
| PCSK9 | rs12732125 | 1 | 55470153 | -0.10344 | 0.007374 | 1.00E-44 | T | C |
| PCSK9 | rs2479395 | 1 | 55484582 | 0.012567 | 0.002218 | 1.50E-08 | C | T |
| PCSK9 | rs77875082* | 1 | 55485042 | 0.048154 | 0.006056 | 1.80E-15 | A | G |
| PCSK9 | rs3976734 | 1 | 55489960 | -0.02975 | 0.002319 | 1.10E-37 | G | A |
| PCSK9 | rs556369867 | 1 | 55491135 | 0.017575 | 0.00243 | 4.80E-13 | T | C |
| PCSK9 | rs200730299* | 1 | 55491853 | -0.05435 | 0.002782 | 5.10E-85 | C | A |
| PCSK9 | rs2479420* | 1 | 55492190 | -0.02839 | 0.002383 | 9.90E-33 | T | C |
| PCSK9 | rs72909541 | 1 | 55494301 | -0.03341 | 0.005015 | 2.70E-11 | T | C |
| PCSK9 | rs17192725 | 1 | 55496131 | 0.030572 | 0.003658 | 6.40E-17 | A | G |
| PCSK9 | rs12739979* | 1 | 55496648 | -0.02026 | 0.00254 | 1.50E-15 | T | C |
| PCSK9 | rs11810371* | 1 | 55496861 | -0.02945 | 0.005073 | 6.40E-09 | A | G |
| PCSK9 | rs72660548* | 1 | 55500978 | 0.050982 | 0.007775 | 5.50E-11 | G | C |
| PCSK9 | rs17111503* | 1 | 55503448 | 0.04068 | 0.002357 | 1.00E-66 | G | A |
| PCSK9 | rs11591147* | 1 | 55505647 | -0.34846 | 0.007931 | 1.00E-200 | T | G |
| PCSK9 | rs11206513 | 1 | 55507649 | 0.031652 | 0.002146 | 3.20E-49 | T | C |
| PCSK9 | rs41294821* | 1 | 55513183 | -0.03866 | 0.007054 | 4.20E-08 | T | C |
| PCSK9 | rs7546522 | 1 | 55516713 | -0.01681 | 0.002953 | 1.20E-08 | T | C |
| PCSK9 | rs2483205 | 1 | 55518316 | -0.02958 | 0.002145 | 2.90E-43 | T | C |
| PCSK9 | rs45613943* | 1 | 55518622 | -0.03407 | 0.004867 | 2.60E-12 | C | T |
| PCSK9 | rs150119739* | 1 | 55520938 | 0.045273 | 0.005202 | 3.20E-18 | A | G |
| PCSK9 | rs472495* | 1 | 55521313 | 0.042574 | 0.002181 | 7.30E-85 | T | G |
| PCSK9 | rs7525503* | 1 | 55522558 | 0.045464 | 0.007582 | 2.00E-09 | T | G |
| PCSK9 | rs11587071 | 1 | 55522674 | -0.02823 | 0.002794 | 5.30E-24 | T | C |
| PCSK9 | rs11206517* | 1 | 55526428 | 0.068029 | 0.005806 | 1.00E-31 | G | T |
| PCSK9 | rs11583974 | 1 | 55551718 | 0.031453 | 0.005171 | 1.20E-09 | A | G |
| PCSK9 | rs56349475* | 1 | 55576102 | -0.0476 | 0.006719 | 1.40E-12 | C | T |
| PCSK9 | rs530804537 | 1 | 55583210 | -0.19234 | 0.009976 | 7.80E-83 | A | G |
| PCSK9 | rs79396670* | 1 | 55588142 | -0.03365 | 0.00562 | 2.10E-09 | A | G |
| HMGCR | rs2006760 | 5 | 74562029 | 0.03556 | 0.002611 | 3.00E-42 | G | C |
| HMGCR | rs115845757 | 5 | 74563700 | 0.048608 | 0.007856 | 6.10E-10 | A | G |
| HMGCR | rs35122945 | 5 | 74610293 | -0.02811 | 0.004238 | 3.30E-11 | C | A |
| HMGCR | rs141642272 | 5 | 74615209 | 0.053282 | 0.00654 | 3.70E-16 | C | G |
| HMGCR | rs111353455 | 5 | 74623949 | 0.024391 | 0.003727 | 6.00E-11 | A | G |
| HMGCR | rs2303152 | 5 | 74641707 | 0.033359 | 0.003453 | 4.40E-22 | A | G |
| HMGCR | rs17648121 | 5 | 74650106 | 0.061985 | 0.006194 | 1.40E-23 | T | C |
| HMGCR | rs55727654 | 5 | 74651864 | 0.042154 | 0.002932 | 6.90E-47 | A | G |
| HMGCR | rs12916 | 5 | 74656539 | 0.062118 | 0.002127 | 1.70E-187 | C | T |
| HMGCR | rs140092661 | 5 | 74682600 | 0.032993 | 0.005822 | 1.50E-08 | T | A |
| HMGCR | rs116153450 | 5 | 74729433 | -0.03036 | 0.004992 | 1.20E-09 | A | C |
| HMGCR | rs116070404 | 5 | 74749969 | -0.02802 | 0.004602 | 1.10E-09 | C | T |

Asterisk (*) represents SNPs selected when the linkage disequilibrium (LD) parameter changes from r^2^<0.3 to r^2^<0.1.

PCSK9, proprotein convertase subtilisin/kexin 9; HMGCR, 3-hydroxy-3-methylglutaryl coenzyme A reductase

**Supplementary Table2** The detail of instrumental variable corresponding to PCSK9 and HMGCR for repeated analysis.

|  | SNP | chr | pos | Beta | SE | *p*-value | Effect alleles | Other alleles |
| --- | --- | --- | --- | --- | --- | --- | --- | --- |
| PCSK9 | rs2479394 | 1 | 55486064 | -0.0386 | 0.0041 | 1.58E-19 | A | G |
| PCSK9 | rs11206510 | 1 | 55496039 | -0.0831 | 0.005 | 2.38E-53 | C | T |
| PCSK9 | rs2495495 | 1 | 55496556 | -0.0342 | 0.0059 | 3.52E-08 | C | T |
| PCSK9 | rs2479409 | 1 | 55504650 | -0.0642 | 0.0041 | 2.51E-50 | A | G |
| PCSK9 | rs11591147 | 1 | 55505647 | -0.497 | 0.018 | 8.57E-143 | T | G |
| PCSK9 | rs4927193 | 1 | 55509872 | -0.0352 | 0.0056 | 4.27E-11 | C | T |
| PCSK9 | rs11206514 | 1 | 55516004 | 0.0507 | 0.0041 | 9.95E-33 | A | C |
| PCSK9 | rs572512 | 1 | 55517344 | 0.0478 | 0.0047 | 5.31E-26 | T | C |
| PCSK9 | rs2495477 | 1 | 55518467 | -0.064 | 0.0054 | 7.28E-30 | G | A |
| PCSK9 | rs585131 | 1 | 55524116 | 0.0637 | 0.005 | 2.70E-35 | T | C |
| PCSK9 | rs12067569 | 1 | 55528629 | 0.0885 | 0.01 | 1.97E-17 | A | G |
| PCSK9 | rs10493176 | 1 | 55538552 | -0.0776 | 0.0102 | 2.54E-14 | G | T |
| PCSK9 | rs11583974 | 1 | 55551718 | 0.0646 | 0.0117 | 3.95E-09 | A | G |
| HMGCR | rs10066707 | 5 | 74560579 | 0.0497 | 0.0054 | 2.97E-19 | A | G |
| HMGCR | rs2006760 | 5 | 74562029 | 0.0533 | 0.0076 | 1.67E-13 | G | C |
| HMGCR | rs72633962 | 5 | 74569028 | 0.06 | 0.0072 | 3.33E-15 | C | T |
| HMGCR | rs10515198 | 5 | 74641560 | 0.0599 | 0.0061 | 5.99E-22 | A | G |
| HMGCR | rs2303152 | 5 | 74641707 | 0.0423 | 0.0064 | 1.04E-09 | A | G |
| HMGCR | rs12916 | 5 | 74656539 | 0.0733 | 0.0038 | 7.79E-78 | C | T |

PCSK9, proprotein convertase subtilisin/kexin 9; HMGCR, 3-hydroxy-3-methylglutaryl coenzyme A reductase

**Supplementary Table3** The effect of PCSK9 and HMGCR inhibitor on autoimmune diseases.

| Outcomes | Drug Target | Method | NSNPs | Beta(95%CI) | OR (95%CI) | *p*-value |
| --- | --- | --- | --- | --- | --- | --- |
| CHD | PCSK9 | MR Egger | 27 | -0.72 (-1.02 to -0.41) | 0.49 (0.36 to 0.66) | 8.96E-05 |
|  |  | Weighted median | 27 | -0.75 (-0.99 to -0.51) | 0.47 (0.37 to 0.60) | 1.02E-09 |
|  |  | Inverse variance weighted | 27 | -0.82 (-0.99 to -0.66) | 0.44 (0.37 to 0.52) | 1.34E-21 |
|  |  | Simple mode | 27 | -0.74 (-1.12 to -0.36) | 0.48 (0.32 to 0.70) | 7.72E-04 |
|  |  | Weighted mode | 27 | -0.73 (-0.98 to -0.48) | 0.48 (0.37 to 0.62) | 5.01E-06 |
|  |  | MR-PRESSO | 27 | -0.82 (-0.99 to -0.66) | 0.44 (0.37 to 0.52) | 5.52E-10 |
|  | HMGCR | MR Egger | 12 | -0.59 (-1.30 to 0.12) | 0.55 (0.27 to 1.12) | 1.33E-01 |
|  |  | Weighted median | 12 | -0.56 (-0.83 to -0.28) | 0.57 (0.43 to 0.75) | 6.86E-05 |
|  |  | Inverse variance weighted | 12 | -0.55 (-0.77 to -0.34) | 0.58 (0.46 to 0.71) | 5.59E-07 |
|  |  | Simple mode | 12 | -0.45 (-0.86 to -0.04) | 0.63 (0.42 to 0.96) | 5.23E-02 |
|  |  | Weighted mode | 12 | -0.53 (-0.79 to -0.27) | 0.59 (0.45 to 0.76) | 1.97E-03 |
|  |  | MR-PRESSO | 12 | -0.55 (-0.73 to -0.38) | 0.58 (0.48 to 0.69) | 7.20E-05 |
| SLE | PCSK9 | MR Egger | 22 | -0.53 (-1.29 to 0.22) | 0.59 (0.28 to 1.25) | 1.82E-01 |
|  |  | Weighted median | 22 | -0.97 (-1.56 to -0.38) | 0.38 (0.21 to 0.68) | 1.21E-03 |
|  |  | Inverse variance weighted | 22 | -0.75 (-1.22 to -0.28) | 0.47 (0.30 to 0.76) | 1.74E-03 |
|  |  | Simple mode | 22 | -1.01 (-1.98 to -0.03) | 0.37 (0.14 to 0.97) | 5.64E-02 |
|  |  | Weighted mode | 22 | -0.81 (-1.37 to -0.25) | 0.45 (0.25 to 0.78) | 1.04E-02 |
|  |  | MR-PRESSO | 22 | -0.79 (-1.27 to -0.3) | 0.46 (0.28 to 0.74) | 4.14E-03 |
|  | HMGCR | MR Egger | 8 | 0.16 (-2.16 to 2.48) | 1.17 (0.12 to 11.89) | 8.97E-01 |
|  |  | Weighted median | 8 | -0.31 (-1.03 to 0.40) | 0.73 (0.36 to 1.49) | 3.90E-01 |
|  |  | Inverse variance weighted | 8 | -0.27 (-0.99 to 0.45) | 0.76 (0.37 to 1.56) | 4.61E-01 |
|  |  | Simple mode | 8 | -0.06 (-1.71 to 1.59) | 0.94 (0.18 to 4.91) | 9.46E-01 |
|  |  | Weighted mode | 8 | -0.28 (-1.00 to 0.44) | 0.75 (0.37 to 1.55) | 4.69E-01 |
|  |  | MR-PRESSO | 8 | -0.09 (-0.77 to 0.59) | 0.91 (0.46 to 1.8) | 7.98E-01 |
| MG | PCSK9 | MR Egger | 26 | 0.11 (-0.61 to 0.83) | 1.12 (0.55 to 2.30) | 7.59E-01 |
|  |  | Weighted median | 26 | 0.25 (-0.41 to 0.92) | 1.29 (0.66 to 2.50) | 4.59E-01 |
|  |  | Inverse variance weighted | 26 | 0.22 (-0.28 to 0.71) | 1.24 (0.76 to 2.04) | 3.87E-01 |
|  |  | Simple mode | 26 | -0.33 (-1.45 to 0.79) | 0.72 (0.23 to 2.20) | 5.66E-01 |
|  |  | Weighted mode | 26 | 0.21 (-0.42 to 0.83) | 1.23 (0.66 to 2.29) | 5.23E-01 |
|  |  | MR-PRESSO | 26 | -0.22 (-0.22 to 0.66) | 1.25 (0.80 to 1.93) | 3.37E-01 |
|  | HMGCR | MR Egger | 12 | 0.37 (-2.32 to 3.06) | 1.45 (0.1 to 21.26) | 7.93E-01 |
|  |  | Weighted median | 12 | -0.10 (-1.12 to 0.93) | 0.91 (0.33 to 2.54) | 8.55E-01 |
|  |  | Inverse variance weighted | 12 | -0.02 (-0.85 to 0.81) | 0.98 (0.43 to 2.25) | 9.66E-01 |
|  |  | Simple mode | 12 | -0.10 (-1.56 to 1.36) | 0.91 (0.21 to 3.90) | 8.97E-01 |
|  |  | Weighted mode | 12 | -0.12 (-1.10 to 0.86) | 0.89 (0.33 to 2.36) | 8.15E-01 |
|  |  | MR-PRESSO | 12 | 0.02 (-0.51 to 0.47) | 0.98 (0.60 to 1.60) | 9.43E-01 |
| RA | PCSK9 | MR Egger | 28 | 0.76 (0.09 to 1.43) | 2.14 (1.09 to 4.18) | 3.57E-02 |
|  |  | Weighted median | 28 | 0.38 (0.03 to 0.73) | 1.46 (1.03 to 2.07) | 3.49E-02 |
|  |  | Inverse variance weighted | 28 | 0.20 (-0.09 to 0.50) | 1.23 (0.92 to 1.64) | 1.70E-01 |
|  |  | Simple mode | 28 | 0.28 (-0.26 to 0.81) | 1.32 (0.77 to 2.25) | 3.20E-01 |
|  |  | Weighted mode | 28 | 0.39 (0.02 to 0.77) | 1.48 (1.02 to 2.15) | 5.07E-02 |
|  |  | MR-PRESSO | 28 | 0.20 (-0.09 to 0.50) | 1.23 (0.92 to 1.64) | 1.81E-01 |
|  | HMGCR | MR Egger | 7 | -0.11 (-1.04 to 0.83) | 0.90 (0.35 to 2.29) | 8.33E-01 |
|  |  | Weighted median | 7 | 0.45 (0.11 to 0.78) | 1.56 (1.11 to 2.19) | 9.81E-03 |
|  |  | Inverse variance weighted | 7 | 0.46 (0.17 to 0.74) | 1.58 (1.19 to 2.11) | 1.67E-03 |
|  |  | Simple mode | 7 | 0.87 (0.22 to 1.52) | 2.38 (1.24 to 4.55) | 3.96E-02 |
|  |  | Weighted mode | 7 | 0.33 (-0.03 to 0.70) | 1.39 (0.97 to 2.01) | 1.23E-01 |
|  |  | MR-PRESSO | 7 | 0.45 (0.20 to 0.70) | 1.56 (1.22 to 2.01) | 0.82E-02 |
| MS | PCSK9 | MR Egger | 6 | 0.04 (-0.38 to 0.45) | 1.04 (0.69 to 1.57) | 8.69E-01 |
|  |  | Weighted median | 6 | -0.16 (-0.53 to 0.21) | 0.85 (0.59 to 1.23) | 3.96E-01 |
|  |  | Inverse variance weighted | 6 | -0.19 (-0.51 to 0.13) | 0.83 (0.60 to 1.13) | 2.38E-01 |
|  |  | Simple mode | 6 | -0.08 (-0.81 to 0.64) | 0.92 (0.45 to 1.90) | 8.34E-01 |
|  |  | Weighted mode | 6 | -0.11 (-0.48 to 0.26) | 0.89 (0.62 to 1.29) | 5.80E-01 |
|  |  | MR-PRESSO | 6 | -0.19 (-0.50 to 0.12) | 0.83 (0.60 to 1.13) | 2.85E-01 |
| Asthma | PCSK9 | MR Egger | 32 | 0.02 (-0.14 to 0.18) | 1.02 (0.87 to 1.19) | 7.96E-01 |
|  |  | Weighted median | 32 | 0.10 (-0.03 to 0.22) | 1.10 (0.97 to 1.25) | 1.20E-01 |
|  |  | Inverse variance weighted | 32 | 0.18 (0.06 to 0.29) | 1.19 (1.07 to 1.33) | 1.97E-03 |
|  |  | Simple mode | 32 | 0.18 (-0.04 to 0.41) | 1.20 (0.96 to 1.50) | 1.18E-01 |
|  |  | Weighted mode | 32 | 0.12 (0.01 to 0.24) | 1.13 (1.01 to 1.27) | 4.50E-02 |
|  |  | MR-PRESSO | 32 | 0.18 (0.06 to 0.29) | 1.19 (1.07 to 1.33) | 4.16E-03 |
|  | PCSK9* | MR Egger | 18 | 0.06 (-0.11 to 0.23) | 1.06 (0.90 to 1.26) | 4.77E-01 |
|  |  | Weighted median | 18 | 0.12 (-0.00 to 0.25) | 1.13 (1.00 to 1.28) | 5.64E-02 |
|  |  | Inverse variance weighted | 18 | 0.14 (0.03 to 0.26) | 1.15 (1.03 to 1.29) | 1.68E-02 |
|  |  | Simple mode | 18 | 0.42 (0.11 to 0.74) | 1.53 (1.11 to 2.10) | 1.79E-02 |
|  |  | Weighted mode | 18 | 0.12 (-0.01 to 0.25) | 1.23 (0.99 to 1.28) | 9.49E-02 |
|  |  | MR-PRESSO | 18 | 0.14 (0.03 to 0.26) | 1.15 (1.03 to 1.29) | 2.86E-02 |
|  | HMGCR | MR Egger | 12 | 0.00 (-0.49 to 0.49) | 1.00 (0.61 to 1.63) | 9.97E-01 |
|  |  | Weighted median | 12 | 0.17 (-0.01 to 0.36) | 1.19 (0.99 to 1.44) | 7.05E-02 |
|  |  | Inverse variance weighted | 12 | 0.19 (0.04 to 0.34) | 1.21 (1.04 to 1.40) | 1.17E-02 |
|  |  | Simple mode | 12 | 0.29 (-0.03 to 0.61) | 1.34 (0.97 to 1.84) | 1.01E-01 |
|  |  | Weighted mode | 12 | 0.18 (0.00 to 0.36) | 1.19 (1.00 to 1.43) | 7.80E-02 |
|  |  | MR-PRESSO | 12 | 0.19 (0.04 to 0.34) | 1.21 (1.04 to 1.40) | 2.84E-02 |
| CD | PCSK9 | MR Egger | 25 | 0.46 (0.03 to 0.90) | 1.59 (1.03 to 2.47) | 4.90E-02 |
|  |  | Weighted median | 25 | 0.28 (-0.06 to 0.62) | 1.33 (0.94 to 1.86) | 1.05E-01 |
|  |  | Inverse variance weighted | 25 | 0.32 (0.04 to 0.60) | 1.38 (1.05 to 1.83) | 2.28E-02 |
|  |  | Simple mode | 25 | 0.24 (-0.42 to 0.89) | 1.27 (0.66 to 2.43) | 4.86E-01 |
|  |  | Weighted mode | 25 | 0.28 (-0.05 to 0.62) | 1.32 (0.95 to 1.85) | 1.14E-01 |
|  |  | MR-PRESSO | 25 | 0.46 (0.03 to 0.90) | 1.59 (1.03 to 2.47) | 2.73E-02 |
|  | HMGCR | MR Egger | 9 | 0.78 (-0.47 to 2.04) | 2.19 (0.63 to 7.66) | 2.60E-01 |
|  |  | Weighted median | 9 | 0.50 (0.05 to 0.95) | 1.65 (1.05 to 2.60) | 2.94E-02 |
|  |  | Inverse variance weighted | 9 | 0.47 (0.07 to 0.87) | 1.60 (1.08 to 2.39) | 2.04E-02 |
|  |  | Simple mode | 9 | 0.52 (-0.26 to 1.30) | 1.68 (0.77 to 3.65) | 2.28E-01 |
|  |  | Weighted mode | 9 | 0.48 (0.01 to 0.95) | 1.61 (1.01 to 2.59) | 8.27E-02 |
|  |  | MR-PRESSO | 9 | 0.78 (-0.47 to 2.04) | 2.19 (0.63 to 7.66) | 5.65E-03 |
| UC | PCSK9 | MR Egger | 28 | -0.12 (-0.73 to 0.48) | 0.88 (0.48 to 1.62) | 6.90E-01 |
|  |  | Weighted median | 28 | -0.03 (-0.52 to 0.46) | 0.97 (0.59 to 1.59) | 9.05E-01 |
|  |  | Inverse variance weighted | 28 | 0.06 (-0.30 to 0.42) | 1.07 (0.74 to 1.52) | 7.30E-01 |
|  |  | Simple mode | 28 | 0.21 (-0.57 to 0.99) | 1.23 (0.56 to 2.70) | 6.02E-01 |
|  |  | Weighted mode | 28 | 0.01 (-0.46 to 0.49) | 1.01 (0.63 to 1.63) | 9.52E-01 |
|  |  | MR-PRESSO | 28 | 0.06 (-0.23 to 0.36) | 1.07 (0.79 to 1.43) | 6.77E-01 |
|  | HMGCR | MR Egger | 12 | 1.87 (0.27 to 3.47) | 6.47 (1.31 to 32.02) | 4.50E-02 |
|  |  | Weighted median | 12 | 0.12 (-0.46 to 0.70) | 1.13 (0.63 to 2.02) | 6.77E-01 |
|  |  | Inverse variance weighted | 12 | -0.06 (-0.56 to 0.44) | 0.94 (0.57 to 1.55) | 8.14E-01 |
|  |  | Simple mode | 12 | -0.13 (-1.29 to 1.02) | 0.87 (0.28 to 2.78) | 8.24E-01 |
|  |  | Weighted mode | 12 | 0.09 (-0.54 to 0.71) | 1.09 (0.59 to 2.03) | 7.89E-01 |
|  |  | MR-PRESSO | 12 | -0.06 (-0.56 to 0.44) | 0.94 (0.57 to 1.55) | 8.18E-01 |
| T1D | PCSK9 | MR Egger | 26 | -0.08 (-0.72 to 0.56) | 0.92 (0.49 to 1.75) | 8.08E-01 |
|  |  | Weighted median | 26 | 0.08 (-0.45 to 0.61) | 1.09 (0.64 to 1.84) | 7.58E-01 |
|  |  | Inverse variance weighted | 26 | -0.02 (-0.43 to 0.39) | 0.98 (0.65 to 1.48) | 9.23E-01 |
|  |  | Simple mode | 26 | -0.74 (-1.79 to 0.30) | 0.48 (0.17 to 1.35) | 1.76E-01 |
|  |  | Weighted mode | 26 | 0.08 (-0.42 to 0.59) | 1.09 (0.66 to 1.80) | 7.47E-01 |
|  |  | MR-PRESSO | 26 | -0.02 (-0.43 to 0.39) | 0.98 (0.65 to 1.48) | 9.24E-01 |
|  | HMGCR | MR Egger | 12 | -0.97 (-2.71 to 0.78) | 0.38 (0.07 to 2.17) | 3.02E-01 |
|  |  | Weighted median | 12 | 0.05 (-0.62 to 0.71) | 1.05 (0.54 to 2.03) | 8.89E-01 |
|  |  | Inverse variance weighted | 12 | 0.02 (-0.51 to 0.56) | 1.02 (0.60 to 1.75) | 9.28E-01 |
|  |  | Simple mode | 12 | 0.14 (-1.16 to 1.43) | 1.14 (0.31 to 4.19) | 8.42E-01 |
|  |  | Weighted mode | 12 | 0.09 (-0.63 to 0.80) | 1.09 (0.53 to 2.23) | 8.14E-01 |
|  |  | MR-PRESSO | 12 | 0.02 (-0.49 to 0.53) | 1.02 (0.62 to 1.71) | 9.27E-01 |

Asterisk (*) represents the linkage disequilibrium (LD) parameter in the selection of instrumental variables changes from r^2^<0.3 to r^2^<0.1. NSNP, number of single nucleotide polymorphisms; OR, odds ratio; CI, confidence interval; PCSK9, proprotein convertase subtilisin/kexin 9; HMGCR, 3-hydroxy-3-methylglutaryl coenzyme A reductase; CHD, coronary heart disease; SLE, systemic lupus erythematosus; RA, rheumatoid arthritis; MG, myasthenia gravis; MS, multiple sclerosis; CD, Crohn's disease; UC, ulcerative colitis; T1D, type 1 diabetes.

**Supplementary Table4** The effect of PCSK9 and HMGCR inhibitor on autoimmune diseases for repeated analysis.

| Outcomes | Drug Target | Method | NSNPs | Beta(95%CI) | OR (95%CI) | p-value |
| --- | --- | --- | --- | --- | --- | --- |
| CHD | PCSK9 | MR Egger | 13 | -0.55 (-0.83 to -0.28) | 0.57 (0.44 to 0.76) | 2.39E-03 |
|  |  | Weighted median | 13 | -0.52 (-0.68 to -0.37) | 0.59 (0.51 to 0.69) | 4.85E-11 |
|  |  | Inverse variance weighted | 13 | -0.52 (-0.65 to -0.39) | 0.60 (0.52 to 0.68) | 6.58E-15 |
|  |  | Simple mode | 13 | -0.54 (-0.81 to -0.27) | 0.58 (0.45 to 0.76) | 2.04E-03 |
|  |  | Weighted mode | 13 | -0.52 (-0.73 to -0.31) | 0.60 (0.48 to 0.73) | 3.57E-04 |
|  |  | MR-PRESSO | 13 | -0.52 (-0.65 to -0.39) | 0.60 (0.52 to 0.68) | 4.92E-06 |
|  | HMGCR | MR Egger | 5 | -1.02 (-1.94 to -0.10) | 0.36 (0.14 to 0.91) | 1.19E-01 |
|  |  | Weighted median | 5 | -0.38 (-0.60 to -0.16) | 0.68 (0.55 to 0.85) | 6.85E-04 |
|  |  | Inverse variance weighted | 5 | -0.37 (-0.54 to -0.20) | 0.69 (0.58 to 0.82) | 2.44E-05 |
|  |  | Simple mode | 5 | -0.39 (-0.75 to -0.03) | 0.68 (0.47 to 0.97) | 1.01E-01 |
|  |  | Weighted mode | 5 | -0.47 (-0.72 to -0.22) | 0.62 (0.48 to 0.81) | 2.22E-02 |
|  |  | MR-PRESSO | 5 | -0.36 (-0.49 to -0.22) | 0.70 (0.61 to 0.80) | 3.32E-03 |
| SLE | PCSK9 | MR Egger | 11 | -0.56 (-1.16 to 0.05) | 0.57 (0.31 to 1.05) | 1.05E-01 |
|  |  | Weighted median | 11 | -0.68 (-1.06 to -0.30) | 0.51 (0.35 to 0.74) | 4.15E-04 |
|  |  | Inverse variance weighted | 11 | -0.58 (-0.90 to -0.25) | 0.56 (0.41 to 0.78) | 5.23E-04 |
|  |  | Simple mode | 11 | -0.81 (-1.32 to -0.31) | 0.44 (0.27 to 0.74) | 1.03E-02 |
|  |  | Weighted mode | 11 | -0.73 (-1.12 to -0.34) | 0.48 (0.33 to 0.71) | 4.16E-03 |
|  |  | MR-PRESSO | 11 | -0.58 (-0.90 to -0.25) | 0.56 (0.41 to 0.78) | 6.03E-03 |
|  | HMGCR | MR Egger | 3 | 1.42 (-4.81 to 7.66) | 4.15 (0.01 to 2116.44) | 7.32E-01 |
|  |  | Weighted median | 3 | -0.31 (-0.93 to 0.31) | 0.74 (0.40 to 1.37) | 3.33E-01 |
|  |  | Inverse variance weighted | 3 | -0.37 (-0.93 to 0.20) | 0.69 (0.39 to 1.22) | 2.07E-01 |
|  |  | Simple mode | 3 | -0.22 (-1.07 to 0.63) | 0.80 (0.34 to 1.88) | 6.65E-01 |
|  |  | Weighted mode | 3 | -0.26 (-0.94 to 0.42) | 0.77 (0.39 to 1.52) | 5.30E-01 |
|  |  | MR-PRESSO | 3 | -0.20 (-0.73 to 0.33) | 0.82 (0.48 to 1.39) | 5.10E-01 |
| MG | PCSK9 | MR Egger | 12 | 0.11 (-0.50 to 0.73) | 1.12 (0.60 to 2.07) | 7.29E-01 |
|  |  | Weighted median | 12 | 0.24 (-0.24 to 0.73) | 1.28 (0.79 to 2.07) | 3.20E-02 |
|  |  | Inverse variance weighted | 12 | 0.34 (-0.02 to 0.70) | 1.40 (0.98 to 2.02) | 6.48E-02 |
|  |  | Simple mode | 12 | 0.12 (-0.69 to 0.94) | 1.13 (0.50 to 2.55) | 7.77E-01 |
|  |  | Weighted mode | 12 | 0.20 (-0.34 to 0.74) | 1.22 (0.71 to 2.10) | 4.80E-01 |
|  |  | MR-PRESSO | 12 | 0.34 (0.03 to 0.65) | 1.40 (1.03 to 1.92) | 5.68E-02 |
|  | HMGCR | MR Egger | 5 | 0.58 (-3.00 to 4.16) | 1.78 (0.05 to 64.24) | 7.72E-01 |
|  |  | Weighted median | 5 | -0.07 (-0.86 to 0.71) | 0.93 (0.43 to 2.04) | 8.60E-01 |
|  |  | Inverse variance weighted | 5 | -0.14 (-0.82 to 0.54) | 0.87 (0.44 to 1.72) | 6.90E-01 |
|  |  | Simple mode | 5 | -0.05 (-1.08 to 0.98) | 0.95 (0.34 to 2.67) | 9.29E-01 |
|  |  | Weighted mode | 5 | -0.06 (-1.03 to 0.91) | 0.94 (0.36 to 2.47) | 9.05E-01 |
|  |  | MR-PRESSO | 5 | -0.13 (-0.34 to 0.08) | 0.88 (0.71 to 1.09) | 2.88E-01 |
| RA | PCSK9 | MR Egger | 12 | 0.07 (-0.48 to 0.62) | 1.07 (0.62 to 1.86) | 8.10E-01 |
|  |  | Weighted median | 12 | 0.14 (-0.08 to 0.36) | 1.15 (0.92 to 1.44) | 2.18E-01 |
|  |  | Inverse variance weighted | 12 | 0.20 (0.02 to 0.38) | 1.22 (1.02 to 1.46) | 2.90E-02 |
|  |  | Simple mode | 12 | 0.18 (-0.2 to 0.55) | 1.19 (0.82 to 1.73) | 3.76E-01 |
|  |  | Weighted mode | 12 | 0.16 (-0.14 to 0.46) | 1.18 (0.87 to 1.59) | 3.15E-01 |
|  |  | MR-PRESSO | 12 | 0.20 (0.02 to 0.38) | 1.22 (1.02 to 1.46) | 5.15E-02 |
|  | HMGCR | MR Egger | 5 | 0.67 (-0.55 to 1.89) | 1.95 (0.57 to 6.62) | 3.63E-01 |
|  |  | Weighted median | 5 | 0.22 (-0.04 to 0.48) | 1.24 (0.96 to 1.61) | 9.64E-02 |
|  |  | Inverse variance weighted | 5 | 0.20 (-0.03 to 0.42) | 1.22 (0.97 to 1.52) | 8.38E-02 |
|  |  | Simple mode | 5 | 0.19 (-0.18 to 0.55) | 1.21 (0.84 to 1.74) | 3.67E-01 |
|  |  | Weighted mode | 5 | 0.24 (-0.04 to 0.52) | 1.27 (0.96 to 1.68) | 1.71E-01 |
|  |  | MR-PRESSO | 5 | 0.25 (0.09 to 0.41) | 1.28 (1.09 to 1.50) | 2.98E-02 |
| MS | PCSK9 | MR Egger | 5 | 0.00 (-0.30 to 0.30) | 1.00 (0.74 to 1.35) | 9.82E-01 |
|  |  | Weighted median | 5 | -0.15 (-0.39 to 0.10) | 0.86 (0.68 to 1.10) | 2.43E-01 |
|  |  | Inverse variance weighted | 5 | -0.18 (-0.42 to 0.05) | 0.83 (0.66 to 1.05) | 1.22E-01 |
|  |  | Simple mode | 5 | -0.23 (-0.60 to 0.13) | 0.79 (0.55 to 1.14) | 2.83E-01 |
|  |  | Weighted mode | 5 | -0.12 (-0.37 to 0.14) | 0.89 (0.69 to 1.14) | 4.14E-01 |
|  |  | MR-PRESSO | 5 | -0.18 (-0.42 to 0.05) | 0.83 (0.66 to 1.05) | 1.97E-01 |
| Asthma | PCSK9 | MR Egger | 13 | 0.08 (-0.07 to 0.24) | 1.09 (0.93 to 1.27) | 3.06E-01 |
|  |  | Weighted median | 13 | 0.07 (-0.01 to 0.15) | 1.07 (0.99 to 1.16) | 9.73E-02 |
|  |  | Inverse variance weighted | 13 | 0.04 (-0.05 to 0.13) | 1.04 (0.95 to 1.14) | 3.74E-01 |
|  |  | Simple mode | 13 | 0.06 (-0.04 to 0.17) | 1.06 (0.96 to 1.18) | 2.82E-01 |
|  |  | Weighted mode | 13 | 0.07 (-0.01 to 0.16) | 1.08 (0.99 to 1.17) | 1.16E-01 |
|  |  | MR-PRESSO | 13 | 0.04 (-0.05 to 0.13) | 1.04 (0.95 to 1.14) | 3.91E-01 |
|  | HMGCR | MR Egger | 5 | 0.02 (-0.64 to 0.68) | 1.02 (0.53 to 1.97) | 9.52E-01 |
|  |  | Weighted median | 5 | 0.12 (-0.03 to 0.27) | 1.13 (0.97 to 1.31) | 1.06E-01 |
|  |  | Inverse variance weighted | 5 | 0.10 (-0.02 to 0.22) | 1.10 (0.98 to 1.25) | 1.17E-01 |
|  |  | Simple mode | 5 | 0.12 (-0.07 to 0.32) | 1.13 (0.93 to 1.38) | 2.86E-01 |
|  |  | Weighted mode | 5 | 0.12 (-0.04 to 0.29) | 1.13 (0.96 to 1.33) | 2.20E-01 |
|  |  | MR-PRESSO | 5 | 0.13 (0.03 to 0.23) | 1.14 (1.03 to 1.26) | 5.28E-02 |
| CD | PCSK9 | MR Egger | 13 | 0.24 (-0.05 to 0.54) | 1.28 (0.95 to 1.72) | 1.37E-01 |
|  |  | Weighted median | 13 | 0.16 (-0.06 to 0.38) | 1.17 (0.94 to 1.46) | 1.55E-01 |
|  |  | Inverse variance weighted | 13 | 0.13 (-0.04 to 0.30) | 1.14 (0.96 to 1.34) | 1.37E-01 |
|  |  | Simple mode | 13 | 0.11 (-0.17 to 0.40) | 1.12 (0.84 to 1.49) | 4.62E-01 |
|  |  | Weighted mode | 13 | 0.16 (-0.07 to 0.39) | 1.17 (0.93 to 1.47) | 2.05E-01 |
|  |  | MR-PRESSO | 13 | 0.13 (0.04 to 0.22) | 1.14 (1.04 to 1.24) | 1.58E-02 |
|  | HMGCR | MR Egger | 5 | 0.54 (-1.06 to 2.14) | 1.71 (0.35 to 8.48) | 5.56E-01 |
|  |  | Weighted median | 5 | 0.35 (0.00 to 0.70) | 1.42 (1.00 to 2.02) | 5.25E-02 |
|  |  | Inverse variance weighted | 5 | 0.34 (0.04 to 0.64) | 1.40 (1.04 to 1.89) | 2.62E-02 |
|  |  | Simple mode | 5 | 0.31 (-0.16 to 0.79) | 1.37 (0.85 to 2.19) | 2.67E-01 |
|  |  | Weighted mode | 5 | 0.37 (-0.06 to 0.81) | 1.45 (0.94 to 2.24) | 1.69E-01 |
|  |  | MR-PRESSO | 5 | -0.37 (-0.55 to -0.18) | 0.69 (0.57 to 0.83) | 1.14E-02 |
| UC | PCSK9 | MR Egger | 13 | -0.02 (-0.48 to 0.44) | 0.98 (0.62 to 1.56) | 9.35E-01 |
|  |  | Weighted median | 13 | -0.02 (-0.34 to 0.29) | 0.98 (0.71 to 1.34) | 8.90E-01 |
|  |  | Inverse variance weighted | 13 | -0.04 (-0.27 to 0.20) | 0.96 (0.76 to 1.22) | 7.64E-01 |
|  |  | Simple mode | 13 | 0.03 (-0.40 to 0.47) | 1.04 (0.67 to 1.61) | 8.80E-01 |
|  |  | Weighted mode | 13 | 0.00 (-0.38 to 0.37) | 1.00 (0.69 to 1.45) | 9.84E-01 |
|  |  | MR-PRESSO | 13 | -0.04 (-0.23 to 0.16) | 0.96 (0.79 to 1.17) | 7.22E-01 |
|  | HMGCR | MR Egger | 5 | 0.54 (-1.06 to 2.14) | 1.71 (0.35 to 8.48) | 5.56E-01 |
|  |  | Weighted median | 5 | 0.35 (0.00 to 0.70) | 1.42 (1.00 to 2.02) | 5.25E-02 |
|  |  | Inverse variance weighted | 5 | 0.34 (0.04 to 0.64) | 1.40 (1.04 to 1.89) | 2.62E-02 |
|  |  | Simple mode | 5 | 0.31 (-0.16 to 0.79) | 1.37 (0.85 to 2.19) | 2.67E-01 |
|  |  | Weighted mode | 5 | 0.37 (-0.06 to 0.81) | 1.45 (0.94 to 2.24) | 1.69E-01 |
|  |  | MR-PRESSO | 5 | 0.16 (-0.33 to 0.65) | 1.18 (0.72 to 1.92) | 1.14E-02 |
| T1D | PCSK9 | MR Egger | 13 | 0.01 (-0.40 to 0.43) | 1.01 (0.67 to 1.54) | 9.50E-01 |
|  |  | Weighted median | 13 | 0.07 (-0.26 to 0.39) | 1.07 (0.77 to 1.48) | 6.79E-01 |
|  |  | Inverse variance weighted | 13 | 0.14 (-0.10 to 0.38) | 1.15 (0.91 to 1.46) | 2.50E-01 |
|  |  | Simple mode | 13 | 0.14 (-0.35 to 0.62) | 1.14 (0.70 to 1.86) | 5.98E-01 |
|  |  | Weighted mode | 13 | 0.05 (-0.30 to 0.40) | 1.05 (0.74 to 1.49) | 7.81E-01 |
|  |  | MR-PRESSO | 13 | 0.14 (-0.06 to 0.34) | 1.15 (0.94 to 1.41) | 1.99E-01 |
|  | HMGCR | MR Egger | 5 | -0.54 (-2.92 to 1.84) | 0.58 (0.05 to 6.28) | 6.86E-01 |
|  |  | Weighted median | 5 | -0.04 (-0.56 to 0.49) | 0.97 (0.57 to 1.64) | 8.95E-01 |
|  |  | Inverse variance weighted | 5 | 0.20 (-0.24 to 0.65) | 1.23 (0.79 to 1.91) | 3.64E-01 |
|  |  | Simple mode | 5 | -0.05 (-0.84 to 0.74) | 0.95 (0.43 to 2.10) | 9.07E-01 |
|  |  | Weighted mode | 5 | -0.04 (-0.68 to 0.60) | 0.96 (0.51 to 1.82) | 9.07E-01 |
|  |  | MR-PRESSO | 5 | -0.24 (-0.53 to 0.05) | 0.79 (0.59 to 1.05) | 1.60E-01 |

NSNP, number of single nucleotide polymorphisms; OR, odds ratio; CI, confidence interval; PCSK9, proprotein convertase subtilisin/kexin 9; HMGCR, 3-hydroxy-3-methylglutaryl coenzyme A reductase; CHD, coronary heart disease; SLE, systemic lupus erythematosus; RA, rheumatoid arthritis; MG, myasthenia gravis; MS, multiple sclerosis; CD, Crohn's disease; UC, ulcerative colitis; T1D, type 1 diabetes.

**Supplementary Table5** The result of heterogeneity test and horizontal pleiotropic test.

| Outcomes | Drug Target | Heterogeneity test | | | | Horizontal pleiotropic test | | |
| --- | --- | --- | --- | --- | --- | --- | --- | --- |
| CHD | PCSK9 | Method | Q | Q_df | Q_pval | egger_intercept | SE | *p*-value |
|  |  | MR Egger | 25.66321 | 25 | 0.4257419 | 0.0048801 | 0.005694 | 0.3995491 |
|  |  | Inverse variance weighted | 26.41726 | 26 | 0.4403628 |  |  |  |
|  | HMGCR | MR Egger | 7.270012 | 10 | 0.699732 | -0.001894 | 0.0163614 | 0.9101565 |
|  |  | Inverse variance weighted | 7.283405 | 11 | 0.7756903 |  |  |  |
| SLE | PCSK9 | MR Egger | 25.38034 | 20 | 0.1872657 | 0.0113688 | 0.0158044 | 0.4802487 |
|  |  | Inverse variance weighted | 26.03701 | 21 | 0.2050415 |  |  |  |
|  | HMGCR | MR Egger | 7.433738 | 6 | 0.2825894 | 0.0229371 | 0.0596297 | 0.7137548 |
|  |  | Inverse variance weighted | 7.617057 | 7 | 0.3675685 |  |  |  |
| MG | PCSK9 | MR Egger | 19.38651 | 24 | 0.731075 | -0.006557 | 0.0166633 | 0.697427 |
|  |  | Inverse variance weighted | 19.54135 | 25 | 0.7704517 |  |  |  |
|  | HMGCR | MR Egger | 3.732204 | 10 | 0.9586202 | 0.0181093 | 0.0608567 | 0.7721197 |
|  |  | Inverse variance weighted | 3.820753 | 11 | 0.9748699 |  |  |  |
| RA | PCSK9 | MR Egger | 33.68625 | 26 | 0.1431075 | 0.0206104 | 0.0115675 | 0.0864752 |
|  |  | Inverse variance weighted | 37.79938 | 27 | 0.0810864 |  |  |  |
|  | HMGCR | MR Egger | 1.691318 | 5 | 0.8899917 | -0.027785 | 0.0223167 | 0.2682891 |
|  |  | Inverse variance weighted | 3.241423 | 6 | 0.7779924 |  |  |  |
| MS | PCSK9 | MR Egger | 1.977899 | 4 | 0.7398241 | 0.0225201 | 0.013244 | 0.1642776 |
|  |  | Inverse variance weighted | 4.869264 | 5 | 0.432043 |  |  |  |
| Asthma | PCSK9 | MR Egger | 45.30736 | 30 | 0.036133 | 0.008893116 | 0.003441 | 0.014867 |
|  |  | Inverse variance weighted | 55.39421 | 31 | 0.004528 |  |  |  |
|  | PCSK9* | MR Egger | 21.08434 | 16 | 0.1752914 | 0.005699902 | 0.00465657 | 0.2386506 |
|  |  | Inverse variance weighted | 23.05877 | 17 | 0.1473471 |  |  |  |
|  | HMGCR | MR Egger | 10.53338 | 10 | 0.395007 | 0.008900432 | 0.011073 | 0.440218 |
|  |  | Inverse variance weighted | 11.21389 | 11 | 0.425523 |  |  |  |
| CD | PCSK9 | MR Egger | 28.75352 | 23 | 0.188621 | -0.007517813 | 0.00917 | 0.420732 |
|  |  | Inverse variance weighted | 29.59376 | 24 | 0.198583 |  |  |  |
|  | HMGCR | MR Egger | 5.87432 | 7 | 0.554499 | -0.015408931 | 0.029921 | 0.62242 |
|  |  | Inverse variance weighted | 6.139532 | 8 | 0.631606 |  |  |  |
| UC | PCSK9 | MR Egger | 17.55038 | 26 | 0.891514 | 0.008930706 | 0.011892 | 0.459409 |
|  |  | Inverse variance weighted | 18.11435 | 27 | 0.899986 |  |  |  |
|  | HMGCR | MR Egger | 5.133683 | 10 | 0.882071 | -0.090597253 | 0.036501 | 0.032429 |
|  |  | Inverse variance weighted | 11.29421 | 11 | 0.418955 |  |  |  |
| T1D | PCSK9 | MR Egger | 32.3438 | 24 | 0.118694 | 0.003463778 | 0.014239 | 0.809875 |
|  |  | Inverse variance weighted | 32.42354 | 25 | 0.146119 |  |  |  |
|  | HMGCR | MR Egger | 8.659612 | 10 | 0.564683 | 0.04648423 | 0.039687 | 0.268642 |
|  |  | Inverse variance weighted | 10.03149 | 11 | 0.527557 |  |  |  |

Asterisk (*) represents the linkage disequilibrium (LD) parameter in the selection of instrumental variables changes from r^2^<0.3 to r^2^<0.1. PCSK9, proprotein convertase subtilisin/kexin 9; HMGCR, 3-hydroxy-3-methylglutaryl coenzyme A reductase; CHD, coronary heart disease; SLE, systemic lupus erythematosus; RA, rheumatoid arthritis; MG, myasthenia gravis; MS, multiple sclerosis; CD, Crohn's disease; UC, ulcerative colitis; T1D, type 1 diabetes.

**Supplementary Table6** The result of heterogeneity test and horizontal pleiotropic test for repeated analysis.

| Outcomes | Drug Target | Heterogeneity test | | | | Horizontal pleiotropic test | | |
| --- | --- | --- | --- | --- | --- | --- | --- | --- |
| CHD | PCSK9 | Method | Q | Q_df | Q_pval | egger_intercept | SE | *p*-value |
|  |  | MR Egger | 16.89899 | 11 | 0.1108992 | -0.002818704 | 0.0093094 | 0.7677071 |
|  |  | Inverse variance weighted | 17.03983 | 12 | 0.1481053 |  |  |  |
|  | HMGCR | MR Egger | 1.38392 | 3 | 0.7093082 | -0.03950181 | 0.0281328 | 0.2549023 |
|  |  | Inverse variance weighted | 3.35547 | 4 | 0.5001918 |  |  |  |
| SLE | PCSK9 | MR Egger | 13.2127 | 9 | 0.1532157 | 0.00184899 | 0.0224772 | 0.9362398 |
|  |  | Inverse variance weighted | 13.22263 | 10 | 0.2114893 |  |  |  |
|  | HMGCR | MR Egger | 0.4696012 | 1 | 0.4931707 | 0.1241546 | 0.2199321 | 0.6728299 |
|  |  | Inverse variance weighted | 0.7882765 | 2 | 0.6742608 |  |  |  |
| MG | PCSK9 | MR Egger | 7.481202 | 10 | 0.6793684 | -0.02081186 | 0.0232351 | 0.3914592 |
|  |  | Inverse variance weighted | 8.283497 | 11 | 0.687706 |  |  |  |
|  | HMGCR | MR Egger | 0.4023503 | 3 | 0.9397566 | 0.04361145 | 0.1089954 | 0.7158544 |
|  |  | Inverse variance weighted | 0.5624478 | 4 | 0.9671438 |  |  |  |
| RA | PCSK9 | MR Egger | 13.04216 | 10 | 0.2213241 | -0.008229386 | 0.016458 | 0.6278777 |
|  |  | Inverse variance weighted | 13.36825 | 11 | 0.2699398 |  |  |  |
|  | HMGCR | MR Egger | 0.7817479 | 3 | 0.8538275 | 0.0293525 | 0.0382464 | 0.498681 |
|  |  | Inverse variance weighted | 1.3707391 | 4 | 0.8492644 |  |  |  |
| MS | PCSK9 | MR Egger | 2.482828 | 3 | 0.4784025 | -0.0221894 | 0.0132355 | 0.1922314 |
|  |  | Inverse variance weighted | 5.293517 | 4 | 0.2584845 |  |  |  |
| Asthma | PCSK9 | MR Egger | 2.520861 | 11 | 0.995666 | -0.010112109 | 0.010958 | 0.375921 |
|  |  | Inverse variance weighted | 3.372378 | 12 | 0.992295 |  |  |  |
|  | HMGCR | MR Egger | 1.887384 | 3 | 0.596106 | -0.012217072 | 0.048878 | 0.818763 |
|  |  | Inverse variance weighted | 1.94986 | 4 | 0.744981 |  |  |  |
| CD | PCSK9 | MR Egger | 4.327988 | 11 | 0.959323 | -0.02153453 | 0.018043 | 0.25777 |
|  |  | Inverse variance weighted | 5.752486 | 12 | 0.928041 |  |  |  |
|  | HMGCR | MR Egger | 0.507008 | 3 | 0.917349 | -0.08333285 | 0.069314 | 0.315507 |
|  |  | Inverse variance weighted | 1.952422 | 4 | 0.74451 |  |  |  |
| UC | PCSK9 | MR Egger | 8.11643 | 11 | 0.702838 | -0.001336165 | 0.016185 | 0.935688 |
|  |  | Inverse variance weighted | 8.123245 | 12 | 0.775426 |  |  |  |
|  | HMGCR | MR Egger | 7.741782 | 3 | 0.051661 | -0.044682502 | 0.103449 | 0.694955 |
|  |  | Inverse variance weighted | 8.223222 | 4 | 0.083735 |  |  |  |
| T1D | PCSK9 | MR Egger | 8.051195 | 11 | 0.708711 | 0.01150284 | 0.015552 | 0.475023 |
|  |  | Inverse variance weighted | 8.598223 | 12 | 0.73681 |  |  |  |
|  | HMGCR | MR Egger | 1.873175 | 3 | 0.599142 | 0.0452611 | 0.072496 | 0.576668 |
|  |  | Inverse variance weighted | 2.262962 | 4 | 0.68752 |  |  |  |

PCSK9, proprotein convertase subtilisin/kexin 9; HMGCR, 3-hydroxy-3-methylglutaryl coenzyme A reductase; CHD, coronary heart disease; SLE, systemic lupus erythematosus; RA, rheumatoid arthritis; MG, myasthenia gravis; MS, multiple sclerosis; CD, Crohn's disease; UC, ulcerative colitis; T1D, type 1 diabetes.
